# Joint Sensing and Secure Communications in RIS-Based Symbiotic Radio Systems

**DOI:** 10.3390/e28020245

**Published:** 2026-02-20

**Authors:** Junhong Yang, Ke-Wen Huang

**Affiliations:** College of Computer Science and Electronic Engineering, Hunan University, Changsha 410082, China; yjh0907@hnu.edu.cn

**Keywords:** dual-functional radar and communication, integrated sensing and communication, reconfigurable intelligent surface, symbiotic radio, secure communications

## Abstract

We study the problem of joint sensing and secure communications in a reconfigurable intelligent surface (RIS)-based symbiotic radio (SR) system. In the considered system, a dual-functional radar and communication base station (DFRC-BS) achieves secure communications with multiple user terminals (UTs), and at the same time, performs a target sensing task. An RIS simultaneously assists the secure communications between the DFRC-BS and the multiple UTs and conveys its own data to the UTs by modulating the radio frequency signal from the DFRC-BS. Two different SR settings are investigated, namely, parasitic SR (PSR) and commensal SR (CSR). In both the PSR and the CSR situations, the echo signal from the sensing target is interfered by the backscattered signal from the RIS. We propose two strategies for the DFRC-BS to handle with the interference from the RIS, namely, (1) directly sensing without interference cancelation, and (2) performing interference cancelation before sensing. For both the two strategies, we aim to maximize the sum secrecy rate from the DFRC-BS to the multiple UTs while ensuring satisfactory performances for the sensing and the backscatter links. A block coordinate ascend algorithm is proposed to solve the established non-convex optimization problems. Simulation results reveal that at the DFRC-BS, performing interference cancelation leads to an improved system performance. Furthermore, compared with PSR, CSR leads to a higher sum secrecy rate between the DFRC-BS and the UTs.

## 1. Introduction

Symbiotic radio (SR), as a potential solution for the future internet of things (IoT) networks, can effectively improve wireless spectral and energy efficiencies [1,2]. In SR systems, backscatter communications-enabled secondary devices are allowed to achieve data transmissions by modulating the radio frequency (RF) signal from other primary transceivers. More importantly, the backscattered signals act as additional signal propagation components that enhance the communications performance between primary transceivers, leading to a mutually beneficial and win–win cooperation mechanism between the primary and the backscatter links in SR systems. Recently, to further enhance the performance of SR systems, a number of studies have proposed to deeply combine the technology of a reconfigurable intelligent surface (RIS) with SR. In brief, an RIS usually exhibits a planar structure composed of a large number of small, low-cost, and passive reflection units (RUs), each of which can induce a phase shift on the reflected electromagnetic wave [3]. By carefully designing the phase shifts of the reflection coefficients, also known as passive beamforming, an RIS can effectively enhance the strength of the reflected signal. In RIS-based SR (RIS-SR) systems, the RISs not only assist the primary transmissions but also act as backscatter devices. Thanks to the large number of the RUs, compared with conventional SR, RIS-SR is shown to achieve a much better performance [4,5,6].

With the emergences of many new IoT application scenarios, such as industrial IoT [7], the internet of drone things [8], and vehicle to everything [9], wireless systems not only need to achieve data transmissions, but also require the ability to perform environmental or target sensing. This has spurred in-depth research on technology of integrated sensing and communication (ISAC)—see, e.g., ref. [10,11,12]. For instance, in [10,11], beam pattern matching problems have been studied to improve the sensing performance while ensuring a satisfactory communication performance. In [12], for a multiple-user multiple-input and multiple-output ISAC system, the authors characterized the achievable performance region for joint sensing and communications.

In ISAC systems, a collaborative operation of sensing and communication can be achieved by sharing hardware and spectrum resources, which effectively improves the spectral efficiency and significantly reduces the system costs [13,14]. Based on this, with the aim of achieving the dual function of sensing and communication while boosting the spectral efficiency, a number of recent studies have introduced the technology of ISAC to SR and RIS-SR systems, which we refer to as SR-ISAC and RIS-SR-ISAC systems, respectively. Specifically, in [15], the authors explored a baggage localization problem in the downlink RIS-SR-ISAC system, and designed a direction of arrival (DoA)-based detector to detect the symbols transmitted over both the primary and the backscatter links. In [16], the primary transmission rate of an RIS-SR-ISAC system was maximized under a group of quality-of-service requirements on the backscatter and the sensing links. The authors of [17] further studied the problem of enhancing the symbiotic transmission performance of RIS-SR-ISAC systems that operate in near-field environments, wherein dynamic metasurface antennas are exploited to improve both the communication and the sensing performances.

Though the studies in [15,16,17] have revealed the superiority of RIS-SR-ISAC, the security-related issues are left unexplored. Due to the openness of wireless systems, wireless data transmissions are vulnerable to interception, eavesdropping, and interference, posing a considerable degree of security threat [18,19,20]. It is worth pointing out that the introduction of ISAC to RIS-SR systems may be beneficial to the security of the wireless data, but new issues are also brought with that. Specifically, in RIS-SR-ISAC systems, the sensing signal can be exploited as artificial noise (AN) which degrades the decoding capability of illegitimate eavesdroppers, and therefore enhances the security of the transmitted data. However, due to the presence of the RIS-based backscatter link, the signal reflected by the RIS acts as a clutter signal which distorts the echo signal reflected by the sensing target, leading to a degraded sensing performance. As a result, a careful design of the signaling scheme is required to simultaneously ensure the security of the data transmission links and the reliability of the sensing links. A thorough evaluation on the system performance is also necessary, which motivates the study in this work.

### Related Works and Motivations

To combat with illegitimate eavesdropping attack, a number of research efforts have been devoted to designing secure transmission schemes for SR/RIS-SR systems—see, e.g., ref. [21,22,23,24,25]. The studies in [21,22] proposed the use of AN for protecting the security of SR systems, and with the aid of elaborated power allocation schemes, the secrecy rates of the SR systems were effectively enhanced. In [23], a cooperative jamming scheme, which is based on two RISs, was designed to improve the secrecy rate of RIS-SR systems. Specifically, in the scheme proposed in [23], one RIS is used to assist in symbiotic communication, while the other one serves as a cooperative jammer that strategically adjusts its reflection coefficient to convert the useful signal into an interfering signal that disrupts the reception at an illegitimate eavesdropper. In [24], an elastic secure transmission framework was proposed for RIS-SR systems, and an effective deep deterministic policy gradient (DDPG) scheme was designed to determine the optimal beamforming. In [25], the authors further improved the security performance of RIS-SR systems by using an active RIS. We note that though these studies have provided effective security solutions, the integration of the sensing function into SR/RIS-SR systems has not been taken into account.

Seve ral existing studies have focused on the issue of data security in ISAC [26,27] and RIS-assisted ISAC (RIS-ISAC) [28,29,30] systems. In [26], a single-user and multiple-target ISAC system was studied, wherein the targets were viewed as potential eavesdroppers. To ensure the security of the transmitted data, the sensing signal was directly exploited as AN to degrade the qualities of the eavesdropping channels. In [27], the authors improved the secure communications performance of multiple-user ISAC networks by leveraging the technique of non-orthogonal multiple access. In [28], an RIS-ISAC system was considered, and the authors proposed to use the high-power radar signal to interfere with the reception at illegitimate eavesdroppers. A joint active and passive beamforming problem was formulated to minimize the eavesdropping signal-to-interference-plus-noise (SINR). In [29], a sensing target was considered to be a potential eavesdropper, and the sensing beam pattern was designed to be maximized towards the target under an information leakage constraint and a group of quality-of-service (QoS) constraints. In [30], the authors studied an active RIS-assisted ISAC system in the presence of an eavesdropper, and the advantages of using an active RIS in improving the security of ISAC systems have been revealed.

We note that in [28,29,30], the RISs are used as pure assistors to enhance the performance of ISAC systems. This is different from RIS-SR systems, wherein the RISs act not only as assistors but also as secondary information sources that convey their own data to intended receivers. By introducing the concept of SR into RIS-ISAC systems, it is possible to further improve some key performance indicators of wireless systems such as spectral efficiency. However, from a security perspective, the secure communications schemes proposed in [23,24,25,28,29,30] cannot be directly applied to RIS-SR-ISAC systems since the RIS-based backscatter links have not been taken into account in these schemes.

Currently, the research on RIS-SR-ISAC systems is still in its infancy. Though the superiority of RIS-SR-ISAC has been revealed in [15,16,17], the security-related issues have been left unexplored. Motivated by the discussions above, in this work, we focus on the secure transmission problem in RIS-SR-ISAC systems. We compare our work with related ones in Table 1, and the main contributions of this work can be summarized as follows:

- We consider an RIS-SR-ISAC system in the presence of an illegitimate eavesdropper (Eve), wherein a dual-functional radar communication base station (DFRC-BS) transmits its confidential data to multiple user terminals (UTs) and simultaneously performs target sensing. An RIS helps improve the secure transmission performance from the DFRC-BS to the UTs, and meanwhile, convey its own data to the UTs via backscatter communications. As far as the authors know, the secure communication performance of such a system has not been evaluated in the literature.
- In the considered system, the sensing link suffers at the interference due to the presence of the backscatter link, and two strategies are considered, namely, (1) sensing without interference cancellation and (2) interference cancellation before sensing. Under both the two strategies, subject to a group of QoS constraints on the backscatter link and a low bound on the sensing SINR, we formulate the problem of maximizing the sum secrecy rate from the DFRC-BS to the UTs. A block coordinate ascend (BCA) algorithm is proposed to handle the established non-convex problems.
- Simulation results are presented to reveal the relationship between the sum secrecy rate and some key system parameters, which reveal that in the considered RIS-SR-ISAC system, having interference at the DFRC-BS can be helpful to increase the sum secrecy rate. Namely, the ICBS strategy outperforms the SWIC strategy. Furthermore, compared with the case where there is no RIS in the considered system, the sum secrecy rate can significantly improved, which demonstrates the mutually beneficial mechanism between the primary and the backscatter links in the considered RIS-SR-ISAC system.

The remainder of this paper is organized as follows. Section 2 introduces the system model and formulates the problems of sum secrecy rate maximization. Section 3 presents the detailed steps of the proposed BCA algorithm. Simulation results are presents in Section 4. And finally, Section 5 concludes the paper.

Notation: ${\left(\cdot \right)}^{T}$, ${\left(\cdot \right)}^{*}$, and ${\left(\cdot \right)}^{H}$ represent the transpose, the conjugate, and the conjugate transpose, respectively. ∥·∥, ${∥\cdot ∥}_{F}$, and Tr(·) denote the $\ell_{2}$-norm, the Frobenius norm, and the trace. diag{x} constructs a diagonal matrix using the entries of vector x, and blkdiag{·} means block diagonal matrix. $C^{M\times N}$ stands for the set of M×N complex vectors or matrices. $\mathrm{CN}(\mu ,\sigma^{2})$ indicates the distribution of complex Gaussian, whose mean and variance are μ and $\sigma^{2}$, respectively. ${\left[x\right]}^{+}$ represents max{x,0}. E[·] means mathematical expectation. X[m,n] denotes the (m,n)-th entry of X. ⊙ represents the Hadamard product. $\lambda_{\max}\left(\cdot \right)$ and $e_{\max}\left(\cdot \right)$ denote the largest eigenvalue and the corresponding eigenvector of a square matrix.

## 2. System Model

**Figure 1:**
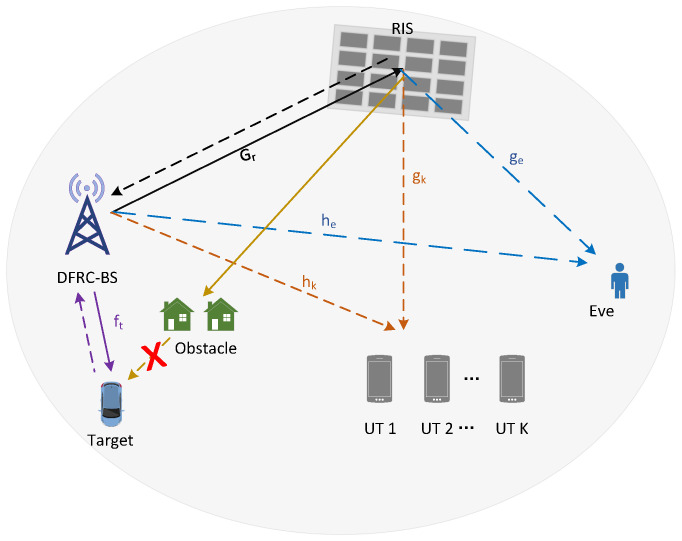
An RIS-based SR system with sensing and communication functionality.

We consider an RIS-based SR system, which consists of a DFRC-BS with *M* antennas, an RIS with *N* reflection units, and *K* single-antenna user terminals (UTs), referred to as UT 1, UT 2, ⋯, and UT *K*, respectively. The DFRC-BS broadcasts sensing signals to perform target sensing, and meanwhile, transmits its data to the *K* UTs. Due to the openness of the wireless environment, the security of the primary links from the DFRC-BS to the *K* UTs is under the threat of a single-antenna eavesdropper (Eve), which aims to obtain the transmit data. The RIS helps in enhancing the security of the primary links, and at the same time, it conveys its own data to the *K* UTs by modulating the RF signal from the DFRC-BS. A comprehensive model is presented in Figure 1.

Denote $f_{t}\in C^{M\times 1}$, $h_{k}\in C^{M\times 1}$, $h_{e}\in C^{M\times 1}$, and $G_{r}\in C^{N\times M}$ as the channels from the DFRC-BS to the sensing target, UT *k*, Eve, and the RIS, respectively. Denote $g_{k}\in C^{N\times 1}$ and $g_{e}\in C^{N\times 1}$ as the channels from the RIS to the UT *k* and Eve, respectively. The channel between the RIS and the target is blocked due to the long link distance and the existence of obstacles [34,35,36]. We assume that the DFRC-BS knows all the channel coefficients. Similar assumptions have been adopted in many existing works [23,30,37]. In fact, the channel coefficients can be well-estimated via a dedicated channel training procedure. For example, by first turning off the RIS and letting the UTs send orthogonal pilot sequences, the direct channels from the UTs to the DFRC-BS can be estimated. Then, by activating one RIS unit at each time and letting the UTs keep sending pilot sequences, the cascaded reflection channels can also be estimated. We note that currently, there have been a number of existing works on the channel estimation problem in RIS assisted wireless systems—see, e.g., ref. [38,39]. And it is reasonable to assume that Eve’s channels are known in the scenario where Eve is an active but distrusted user. We also note that in this work, we assume that the target, the UTs, and Eve are all located in the far-field regions of the multi-antenna DFRC-BS and the RIS. Under this assumption, the electromagnetic waves, when arriving at the target, the UTs, and Eve, can be well approximated as planar waves. It is worth mentioning that such approximations may become invalid in certain scenarios; for example, when the DFRC-BS is equipped with extremely large-scale MIMO [40]. In that case, a spherical wavefront should be considered, and to characterize its effects, a near-field channel model is required, which is, however, beyond the scope of this paper, but constitutes an interesting future issue.

It is worth mentioning that in the considered system, the sensing signal broadcasted by the DFRC-BS can be exploited as AN to enhance the security of the primary links. However, due to the presence of the RIS-based backscatter links, the signal reflected by the RIS could interfere with the echo signal from the target, degrading the sensing performance. In order to ensure a satisfactory system performance, a careful design of the transmit signal of the DFRC-BS and the reflection coefficients of the RIS is necessary.

### 2.1. Signaling Scheme

For a typical data frame consisting of a total number of *T* symbol periods, the signal transmitted by the DFRC-BS at time *q*, q∈{1,2,⋯,T}, is written as   

(1) $$x\left(q\right)=\sum_{k=1}^{K}w_{k}s_{k}\left(q\right)+z_{s}\left(q\right),$$

where $w_{k}\in C^{M\times 1}$ and $s_{k}\left(q\right)$∼CN(0,1) denote the beamforming vector and the information-carrying symbol for UT *k*, $z_{s}\left(q\right)$∼CN(0,Σ) with $\Sigma \in C^{M\times M}$ as the sensing signal. We note that the role of the sensing signal is twofold. On the one hand, it is exploited for target sensing. On the other hand, it interferes with the reception at Eve, enhancing the security of the primary links.

At the RIS, a segmentation scheme is considered to facilitate it to be able to simultaneously assist the secure communications between the DFRC-BS and the *K* UTs and achieve backscatter communications. Specifically, the RIS is segmented into two sub-surfaces, referred to as sub-surface 1 and sub-surface 2. Denote by $N_{1}$ and $N_{2}$ the numbers of the RUs in sub-surface 1 and sub-surface 2, respectively, and we have that $N_{1}+N_{2}=N$. We assume that sub-surface 1 is used to assist the secure transmissions from the DFRC-BS to the *K* UTs, and sub-surface 2 is used for backscatter communications. The reflection matrix of the RIS at time q∈{1,2,⋯,T} can be written as

(2) $$\begin{array}{c} \Phi \left(q\right)=\mathrm{blkdiag}\left\{\Phi_{1},c\left(q\right)\Phi_{2}\right\}\in C^{N\times N} \end{array}$$

where for ∀i∈{1,2}, $\Phi_{i}=\mathrm{diag}\left\{\phi_{i}\right\}\in C^{N_{i}\times N_{i}}$ with $\phi_{i}≜{\left[\phi_{i,1},\phi_{i,2},\cdots ,\phi_{i,N_{i}}\right]}^{T}\in C^{N_{i}\times 1}$, and c(q)∈{−1,1} represents the information-carrying symbol that the RIS desires to send to the *K* UTs at time *q*. Due to the passive nature of the RIS, each reflection coefficient is subject to a constant modulus constraint, namely, $|\phi_{i,j}|\;=1$ for ∀(i,j). We note that such a segmentation scheme has also been adopted in existing works such as [41,42,43,44] for its simplicity and flexibility.

Denote by $y_{k}\left(q\right)$ and $y_{e}\left(q\right)$ the signal received by UT *k* and Eve at time *q*, respectively. Denote by $y_{b}\left(q\right)\in C^{M\times 1}$ the echo signal received by the DFRC-BS at time *q*. According to (1) and (2), we have that

(3) $$\begin{array}{cc} y_{k}\left(q\right) & =\left(h_{k}^{H}+g_{k}^{H}\Phi \left(q\right)G_{r}\right)x\left(q\right)+z_{k}\left(q\right),\;\;\forall k,q, \end{array}$$

(4) $$\begin{array}{cc} y_{e}\left(q\right) & =\left(h_{e}^{H}+g_{e}^{H}\Phi \left(q\right)G_{r}\right)x\left(q\right)+z_{e}\left(q\right),\;\;\forall q, \end{array}$$

(5) $$\begin{array}{cc} y_{b}\left(q\right) & =f_{t}f_{t}^{H}x\left(q\right)+G_{r}^{H}\Phi \left(q\right)G_{r}x\left(q\right)+z_{b}\left(q\right),\;\;\forall q, \end{array}$$

where $z_{k}\left(q\right)∼\mathrm{CN}\left(0,\sigma_{k}^{2}\right)$, $z_{e}\left(q\right)∼\mathrm{CN}\left(0,\sigma_{e}^{2}\right)$, and $z_{b}\left(q\right)∼\mathrm{CN}\left(0,\sigma_{b}^{2}I_{M}\right)$ are the additive white Gaussian noise (AWGN) at UT *k*, Eve, and the DFRC-BS, respectively.

Depending on the symbol period of the backscatter link, SR systems are classified into parasitic SR (PSR) and commensal SR (CSR) systems [45], which usually exhibit different system performance. For the case of PSR, the symbol period of the RIS-based backscatter link keeps the same as that of the primary link, and as a result, {c(1),c(2),⋯,c(T)} is modeled as a sequence of identically and independently distributed random variables. Since c(q), 1≤q≤T, is unknown to the DFRC-BS and the UTs prior, the existence of the RIS-based backscatter link causes interference at both the DFRC-BS and the UTs.

For the case of CSR, the symbol period of the RIS-based backscatter link is *Q* times that of the primary link, where Q≫1. Let i∈{1,2,⋯,⌊T/Q⌋} and $\tilde{q}\in \left\{1,\cdots ,Q\right\}$ be two integers such that $q=\left(i-1\right)Q+\tilde{q}$. Then, in CSR systems, we have that for $\forall \tilde{q}\in \left\{1,\cdots ,Q\right\}$, $c\left(\left(i-1\right)Q+\tilde{q}\right)\equiv c_{i}$, where $c_{i}\in \left\{+1,-1\right\}$ is the *i*-th information-carrying symbol sent by the RIS. Due to the fact that in CSR systems, c(q) keeps unchanged for a relatively long period of time, both the signals reflected by sub-surface 1 and sub-surface 2 of the RIS can provide additional multi-path components for the primary link from the DFRC-BS to the UTs. Due to the different symbol periods of the backscatter links, the PSR and the CSR systems, in general, are suited to different application scenarios. Specifically, the PSR systems are more suitable for situations that requires high backscatter transmission but low primary transmission rates, whereas the CSR systems are more suitable to opposite cases [46].

We discuss separately the performances of the considered system under the PSR and the CSR settings below.

### 2.2. System Performance Under PSR

We reformulate $y_{k}\left(q\right)$ in (3) as

(6) $$\begin{array}{cc} y_{k}\left(q\right)= & f_{k,1}^{H}w_{k}s_{k}\left(q\right)+f_{k,1}^{H}x_{\bar{k}}\left(q\right)\\ \; & \;\;\;\;\;+c\left(q\right)f_{k,2}^{H}x\left(q\right)+z_{k}\left(q\right), \end{array}$$

where for $f_{k,1}≜h_{k}+G_{r,1}^{H}\Phi_{1}^{H}g_{k,1}\in C^{M\times 1}$, $f_{k,2}≜G_{r,2}^{H}\Phi_{2}^{H}g_{k,2}\in C^{M\times 1}$, $g_{k,i}\in C^{N_{i}\times 1}$ denotes the channel between sub-surface *i* and UT *k* which satisfies that $g_{k}={\left[g_{k,1}^{H},g_{k,2}^{H}\right]}^{H}$, $G_{r,i}\in C^{N_{i}\times M}$ denotes the channel between the DFRC-BS and sub-surface *i* which satisfies that $G_{r}={\left[G_{r,1}^{H},G_{r,2}^{H}\right]}^{H}$, and $x_{\bar{k}}\left(q\right)≜\sum_{j\neq k}w_{j}s_{j}\left(q\right)+z_{s}\left(q\right)=x\left(q\right)-w_{k}s_{k}\left(q\right)$.

Since c(q) randomly changes in each symbol period of the primary link, we assume that UT *k* treats it as noise. As a result, the SINR of UT *k* for decoding $s_{k}\left(q\right)$, q∈{1,2,⋯,T}, is obtained as

(7) $$\begin{array}{c} \gamma_{p,k}^{psr}=\frac{|f_{k,1}^{H}w_{k}{|}^{2}}{I_{k}+\sigma_{k}^{2}}, \end{array}$$

where $I_{k}≜|f_{k,2}^{H}w_{k}{|}^{2}+\sum_{i\neq k}\left|\right|F_{k}^{H}w_{i}{\left|\right|}^{2}+\mathrm{Tr}\left\{F_{k}^{H}\Sigma F_{k}\right\}$ with $F_{k}≜\left[f_{k,1},f_{k,2}\right]\in C^{M\times 2}$. Accordingly, an achievable communication rate from the DFRC-BS to UT *k* is given by

(8) $$\begin{array}{cc} R_{k}^{psr} & =\log_{2}\left(1+\gamma_{p,k}^{psr}\right). \end{array}$$

After obtaining $s_{k}\left(q\right)$, UT *k* first performs successive interference cancellation (SIC), and then decodes c(q)  [47,48]. Based on (6), the average SINR for decoding c(q) at UT *k* is given by

(9) $$\begin{array}{c} \gamma_{b,k}^{psr}=\frac{|f_{k,2}^{H}w_{k}{|}^{2}}{I_{k}^{\prime }+\sigma_{k}^{2}} \end{array}$$

where $I_{k}^{\prime }≜\sum_{i\neq k}\left|\right|F_{k}^{H}w_{i}{\left|\right|}^{2}+\mathrm{Tr}\left\{F_{k}^{H}\Sigma F_{k}\right\}$. We assume that in order for the UTs to successfully decode the data from the RIS, the average SINR is required to exceed a pre-given threshold, denoted by $\mu_{th}$. In other words, it is required that $\gamma_{b,k}^{psr}\geq \mu_{th}$ for ∀k.

At the side of Eve, it aims to intercept the data from the DFRC-BS. When Eve decodes the data intended for UT *k*, we consider a worst case scenario wherein the symbols transmitted to other UTs, i.e., $\left\{s_{j}\left(q\right):1\leq j\neq k\leq K,1\leq q\leq T\right\}$, and the symbols transmitted over the RIS-based backscatter link, i.e., {c(q):1≤k≤T}, are known to Eve, and thus do not cause interference [49]. According to (4), an upper bound on the eavesdropping rate when Eve decodes the data of UT *k* is given by

(10) $$\begin{array}{cc} R_{e,k} & =E_{c}\left\{\log_{2}\left(1+\gamma_{e,k}\left(c\right)\right)\right\}\\ \; & =\sum_{j=1}^{2}\frac{1}{2}\log_{2}\left(1+\frac{|f_{e,j}^{H}w_{k}{|}^{2}}{f_{e,j}^{H}\Sigma f_{e,j}+\sigma_{e}^{2}}\right), \end{array}$$

where $\gamma_{e,k}\left(c\right)≜\frac{|f_{e}^{H}\left(c\right)w_{k}{|}^{2}}{f_{e}^{H}\left(c\right)\Sigma f_{e}\left(c\right)+\sigma_{e}^{2}}$, $f_{e}^{H}\left(c\right)≜h_{e}^{H}+g_{e,1}^{H}\Phi_{1}G_{r,1}+cg_{e,2}^{H}\Phi_{2}G_{r,2}$, *c* is a random variable distributed over {1,−1} with equal probability, $f_{e,1}^{H}≜f_{e}^{H}\left(1\right)$, and $f_{e,2}^{H}≜f_{e}^{H}\left(-1\right)$. Consequently, an achievable secrecy rate of UT *k* is obtained as

(11) $$\begin{array}{c} R_{s,k}^{psr}={\left(R_{k}^{psr}-R_{e,k}\right)}^{+}. \end{array}$$

At the side of the DFRC-BS, it performs target sensing using its received signal. We reformulate (5) as

(12) $$\begin{array}{cc} y_{b}\left(q\right)= & F_{t}x\left(q\right)+G_{r,1}^{H}\Phi_{1}G_{r,1}x\left(q\right)\\ \; & +c\left(q\right)G_{r,2}^{H}\Phi_{2}G_{r,2}x\left(q\right)+z_{b}\left(q\right),\forall q, \end{array}$$

where $F_{t}≜f_{t}f_{t}^{H}$. We note that $y_{b}\left(q\right)$ not only contains the echo signal from the target, but also contains the signal reflected by the RIS. Since the DFRC-BS knows x(q), $G_{r}$, and $\Phi_{1}$, it can directly cancel a part of the signal reflected by the RIS, and obtains

(13) $$\begin{array}{c} {\tilde{y}}_{b}\left(q\right)=y_{b}\left(q\right)-G_{r,1}^{H}\Phi_{1}G_{r,1}x\left(q\right)=F_{t}x\left(q\right)+c\left(q\right)G_{r,2}^{H}\Phi_{2}G_{r,2}x\left(q\right)+z_{b}\left(q\right),\forall q. \end{array}$$

Based on ${\tilde{y}}_{b}\left(q\right)$, let $r_{1}\in C^{M\times 1}$ be the receiving beamforming vector for target sensing. Then, the DFRC-BS further obtains

(14) $$\begin{array}{cc} {\dot{y}}_{b}\left(q\right) & =r_{1}^{H}{\tilde{y}}_{b}\left(q\right)\\ \; & =\underset{\mathrm{Desired}\;\mathrm{echo}\;\mathrm{signal}}{\underset{︸}{r_{1}^{H}F_{t}x\left(q\right)}}+\underset{\mathrm{Interference}\;\mathrm{due}\;\mathrm{to}\;\mathrm{the}\;\mathrm{backscatter}\;\mathrm{link}}{\underset{︸}{c\left(q\right)r_{1}^{H}G_{r,2}^{H}\Phi_{2}G_{r,2}x\left(q\right)}}+\underset{\mathrm{AWGN}}{\underset{︸}{r_{1}^{H}z_{b}\left(q\right)}}. \end{array}$$

In (14), ${\dot{y}}_{b}\left(q\right)$ is still impacted by the signal reflected by the RIS because the RIS’s symbols are unknown, i.e., {c(q):1≤q≤T}. We consider two different strategies at the DFRC-BS, depending on how it handles the interfering term in (14), i.e., $c\left(q\right)r_{1}^{H}G_{r,2}^{H}\Phi_{2}G_{r,2}x\left(q\right)$. The first strategy is referred to as sensing without interference cancelation (SWIC). Specifically, the DFRC-BS directly performs target sensing based on ${\dot{y}}_{b}\left(q\right)$ by treating the signal reflected by the RIS as noise. The second strategy is referred to as interference cancelation before sensing (ICBS), in which the DFRC-BS first decodes c(q) using ${\tilde{y}}_{b}\left(q\right)$ and then cancels the interfering term in ${\dot{y}}_{b}\left(q\right)$. After that, it can perform target sensing in the absence of the interference from the RIS. We separately discuss the two strategies below.

#### 2.2.1. SWIC

As mentioned above, in the SWIC strategy, the first term on the right-hand-side of (14) is viewed as the desired echo signal, and the interfering term is treated as a noise. Therefore, the average sensing SINR at the DFRC-BS is given by

(15) $$\gamma_{b,t}^{\mathrm{SWIC}}=\frac{E\{|r_{1}^{H}F_{t}x\left(q\right){|}^{2}\}}{I_{b,t}^{\mathrm{SWIC}}+E\left\{r_{1}^{H}z_{b}\left(q\right)\right\}}=\frac{\sum_{k=1}^{K}{\left|r_{1}^{H}F_{t}w_{k}\right|}^{2}+r_{1}^{H}F_{t}\Sigma F_{t}^{H}r_{1}}{I_{b,t}^{\mathrm{SWIC}}+\left|\right|r_{1}{\left|\right|}^{2}\sigma_{b}^{2},}$$

where $I_{b,t}^{\mathrm{SWIC}}$ is the power of the interference from the RIS, which is calculated as

(16) $$\begin{array}{cc} I_{b,t}^{\mathrm{SWIC}} & =E\left\{{\left|c\left(q\right)r_{1}^{H}G_{r,2}^{H}\Phi_{2}G_{r,2}x\left(q\right)\right|}^{2}\right\}\\ \; & =\sum_{k=1}^{K}\left({\left|r_{1}^{H}G_{r,2}^{H}\Phi_{2}G_{r,2}w_{k}\right|}^{2}\right)\\ \; & \;+r_{1}^{H}G_{r,2}^{H}\Phi_{2}G_{r,2}\Sigma G_{r,2}^{H}\Phi_{2}^{H}G_{r,2}r_{1}. \end{array}$$

#### 2.2.2. ICBS

In the ICBS strategy, the DFRC-BS first decodes c(q) after obtaining ${\tilde{y}}_{b}\left(q\right)$ by using a receiving beamforming vector, denoted by $r_{2}\in C^{M\times 1}$. Specifically, the DFRC-BS obtains

(17) $$\begin{array}{cc} {\bar{y}}_{b}\left(q\right)=r_{2}^{H}{\tilde{y}}_{b}\left(q\right)= & c\left(q\right)r_{2}^{H}G_{r,2}^{H}\Phi_{2}G_{r,2}x\left(q\right)\\ \; & +r_{2}^{H}F_{t}x\left(q\right)+r_{2}^{H}z_{b}. \end{array}$$

Accordingly, the average SINR for decoding c(q) at the DFRC-BS can be given by

(18) $$\gamma_{b,r}^{psr}=\frac{r_{2}^{H}G_{r,2}^{H}\Phi_{2}G_{r,2}\left(WW^{H}+\Sigma \right)G_{r,2}^{H}\Phi_{2}^{H}G_{r,2}r_{2}}{I_{b,t}^{psr}+\left|\right|r_{2}{\left|\right|}^{2}\sigma_{b}^{2}},$$

where $W≜\left[w_{1},w_{2},\cdots ,w_{K}\right]\in C^{M\times K}$, and $I_{b,t}^{psr}≜\sum_{k=1}^{K}{\left|r_{2}^{H}F_{t}w_{k}\right|}^{2}+r_{2}^{H}F_{t}\Sigma F_{t}^{H}r_{2}$. By assumption, the DFRC-BS can successfully decode {c(q):1≤q≤T} only when $\gamma_{b,r}^{psr}\geq \mu_{th}$. And if this is the case, the DFRC-BS can cancel the interference due to the presence of the RIS-based backscatter link in ${\dot{y}}_{b}\left(q\right)$, and obtains

(19) $$\begin{array}{cc} {\ddot{y}}_{b}\left(q\right) & ={\ddot{y}}_{b}\left(q\right)-c\left(q\right)r_{1}^{H}G_{r,2}^{H}\Phi_{2}G_{r,2}x\left(q\right)=r_{1}^{H}F_{t}x\left(q\right)+r_{1}^{H}z_{b}. \end{array}$$

Then, based on ${\ddot{y}}_{b}\left(q\right)$ in (19), the average sensing SINR at the DFRC-BS becomes

(20) $$\begin{array}{c} \gamma_{b,t}^{\mathrm{ICBS}}=\frac{E\{|r_{1}^{H}F_{t}x\left(q\right){|}^{2}\}}{E\{r_{1}^{H}z_{b}\left(q\right)\}}=\frac{\sum_{k=1}^{K}{\left|r_{1}^{H}F_{t}w_{k}\right|}^{2}+r_{1}^{H}F_{t}\Sigma F_{t}^{H}r_{1}}{\left|\right|r_{1}{\left|\right|}^{2}\sigma_{b}^{2}}. \end{array}$$

### 2.3. System Performance Under CSR

Under the CSR case, a single data frame of the DFRC-BS is divided into multiple sub-frames of length *Q*, and in each sub-frame, the information-carrying symbol of the RIS remains unchanged. Let $y_{k,i}\left(\tilde{q}\right)$ be the signal received by UT *k* during the $\tilde{q}$-th symbol period of the *i*-th sub-frame. Then, we have

(21) $$\begin{array}{cc} y_{k,i}\left(\tilde{q}\right)= & y_{k}\left(\left(i-1\right)Q+\tilde{q}\right)\\ = & {\left(f_{k}\left(c_{i}\right)\right)}^{H}w_{k}s_{k,i}\left(\tilde{q}\right)+{\left(f_{k}\left(c_{i}\right)\right)}^{H}x_{\bar{k},i}\left(\tilde{q}\right)+z_{k,i}\left(\tilde{q}\right), \end{array}$$

where $s_{k,i}\left(\tilde{q}\right)≜s_{k}\left(\left(i-1\right)Q+\tilde{q}\right)$, $z_{k,i}\left(\tilde{q}\right)≜z_{k}\left(\left(i-1\right)Q+\tilde{q}\right)$, $x_{\bar{k},i}\left(\tilde{q}\right)=x\left(\left(i-1\right)Q+\tilde{q}\right)-w_{k}s_{k,i}\left(\tilde{q}\right)$, and $f_{k}\left(c_{i}\right)≜f_{k,1}+c_{i}^{*}f_{k,2}$.

It is worth mentioning that under the CSR setting, it is commonly assumed that *Q* is sufficiently large, namely, Q≫1—see, e.g., ref. [45,47,48]. This enables UT *k* to accurately estimate the equivalent channel coefficient, i.e., ${\left(f_{k}\left(c_{i}\right)\right)}^{H}w_{k}$, when decoding the information-carrying symbols from the DFRC-BS, i.e., $\left\{s_{k,i}\left(1\right),s_{k,i}\left(2\right),\cdots ,s_{k,i}\left(Q\right)\right\}$. As a result, if Q≫1, the SINR of the *k*-th user for decoding the $\left\{s_{k,i}\left(\tilde{q}\right):1\leq \tilde{q}\leq Q\right\}$ can be approximated as [45],

(22) $$\begin{array}{c} \gamma_{p,k}^{csr}\left(c_{i}\right)≜\frac{|{\left(f_{k}\left(c_{i}\right)\right)}^{H}w_{k}{|}^{2}}{\left|\right|W_{\bar{k}}^{H}f_{k}\left(c_{i}\right){\left|\right|}^{2}+{\left(f_{k}\left(c_{i}\right)\right)}^{H}\Sigma f_{k}\left(c_{i}\right)+\sigma_{k}^{2}}, \end{array}$$

where $W_{\bar{k}}\in C^{M\times (K-1)}$ is a matrix obtained by deleting the *k*-th column of matrix W. And thus the average information rate from the DFRC-BS to UT *k* can be written as

(23) $$\begin{array}{cc} R_{k}^{csr}= & E_{c}\{\log_{2}\left(1+\gamma_{p,k}^{csr}\left(c_{i}\right)\right\}\\ = & \frac{1}{2}\log_{2}\left(1+\gamma_{p,k}^{csr}\left(1\right)\right)+\frac{1}{2}\log_{2}\left(1+\gamma_{p,k}^{csr}\left(-1\right)\right). \end{array}$$

After decoding the primary symbol from the DFRC-BS, UT *k* can subtract the signal component that propagates thought the direct channel, and obtains

(24) $$\begin{array}{cc} {\tilde{y}}_{k,i}\left(\tilde{q}\right)= & y_{k,i}\left(\tilde{q}\right)-f_{k,1}^{H}w_{k}s_{k,i}\left(\tilde{q}\right)\\ = & c_{i}f_{k,2}^{H}w_{k}s_{k,i}\left(\tilde{q}\right)+{\left(f_{k}\left(c_{i}\right)\right)}^{H}x_{\bar{k},i}\left(\tilde{q}\right)+z_{k,i}\left(\tilde{q}\right),\forall \tilde{q}, \end{array}$$

To obtain $c_{i}$, the first term in the right-hand-side of (24) is viewed as the desired signal and the remaining terms are treated as noise. By combining ${\tilde{y}}_{k,i}\left(1\right),{\tilde{y}}_{k,i}\left(2\right),\cdots ,{\tilde{y}}_{k,i}\left(Q\right)$ together, under the condition that Q≫1, the average SINR for UT *k* to recover $c_{i}$ is given by

(25) $$\gamma_{b,k}^{csr}=\frac{Q|f_{k,2}^{H}w_{k}{|}^{2}}{\left|\right|F_{k}^{H}W_{\bar{k}}{\left|\right|}_{F}^{2}+\mathrm{Tr}\left\{F_{k}^{H}\Sigma F_{k}\right\}+\sigma_{k}^{2}}$$

Similar to the case of PSR, we assume that UT *k* can successfully obtain the data from the RIS if $\gamma_{b,k}^{csr}$ exceeds a pre-given threshold, namely, $\gamma_{b,k}^{csr}\geq \mu_{th}$.

At the side of Eve, it aims to obtain the data that the DFRC-BS transmits to the UTs. Similarly to the PSR setting as discussed in Section 2.2, when Eve decodes $\left\{s_{k}\left(q\right):1\leq q\leq T\right\}$, we assume that it can cancel the signals intended for other UTs, which constitutes a worst case from a security perspective. Therefore, the secrecy rate of UT *k* can be expressed as

(26) $$R_{s,k}^{csr}={\left(R_{k}^{csr}-R_{e,k}\right)}^{+}.$$

where $R_{e,k}$ is given in (10).

At the DFRC-BS, during the $\tilde{q}$-th symbol period of the *i*-th sub-frame, by canceling the interfering signals from the RIS that are known in prior, it obtains

(27) $$\begin{array}{cc} {\tilde{y}}_{b,i}\left(\tilde{q}\right)= & F_{t}x_{i}\left(\tilde{q}\right)+c_{i}G_{r,2}^{H}\Phi_{2}G_{r,2}x_{i}\left(\tilde{q}\right)+z_{b,i}\left(\tilde{q}\right), \end{array}$$

where for $\forall (i,\tilde{q})$, $x_{i}\left(\tilde{q}\right)≜x\left(\left(i-1\right)Q+\tilde{q}\right)$ and $z_{b,i}\left(\tilde{q}\right)≜z_{b}\left(\left(i-1\right)Q+\tilde{q}\right)$. Similar to the case of PSR, the DFRC-BS can adopt either an SWIC or an ICBS strategy.

If the SWIC strategy is adopted, then according to (27), the sensing SINR is actually the same as (15). If the ICBS strategy is adopted, a receiving beamforming vector, denoted by $r_{2}$, is used to recover $c_{i}$ first. Specifically, the DFRC-BS obtains

(28) $$\begin{array}{cc} {\hat{y}}_{b,i}\left(\tilde{q}\right)= & r_{2}^{H}{\tilde{y}}_{b,i}\left(\tilde{q}\right)\\ = & c_{i}{\hat{s}}_{i}\left(\tilde{q}\right)+v_{i}\left(\tilde{q}\right)+{\tilde{z}}_{i}\left(\tilde{q}\right),\;\;\tilde{q}\in \left\{1,2,\cdots ,Q\right\}, \end{array}$$

where ${\hat{s}}_{i}\left(\tilde{q}\right)≜r_{2}^{H}G_{r,2}^{H}\Phi_{2}G_{r,2}x_{i}\left(\tilde{q}\right)$, $v_{i}\left(\tilde{q}\right)≜r_{2}^{H}F_{t}x_{i}\left(\tilde{q}\right)$, and ${\tilde{z}}_{i}\left(\tilde{q}\right)≜r_{2}^{H}z_{b,i}\left(\tilde{q}\right)$. Note that the DFRC-BS can combine the *Q* signals in the *i*-th sub-frame together to obtain $c_{i}$. For example, using an equal gain combiner, the DFRC-BS obtains

(29) $$\begin{array}{cc} {\overset{ˇ}{y}}_{b,i}= & \sum_{\tilde{q}=1}^{Q}e^{-j∠\{{\hat{s}}_{i}\left(\tilde{q}\right)\}}{\hat{y}}_{b,i}\left(\tilde{q}\right)\\ = & c_{i}S_{b,r,i}+I_{b,r,i}+Z_{b,r,i}, \end{array}$$

where $S_{b,r,i}≜\sum_{\tilde{q}=1}^{Q}\left|{\hat{s}}_{i}\left(\tilde{q}\right)\right|$, $I_{b,r,i}≜\sum_{\tilde{q}=1}^{Q}e^{-j∠\{{\hat{s}}_{i}\left(\tilde{q}\right)\}}v_{i}\left(\tilde{q}\right)$, and $Z_{b,r,i}≜\sum_{\tilde{q}=1}^{Q}e^{-j∠\{{\hat{s}}_{i}\left(\tilde{q}\right)\}}{\tilde{z}}_{i}\left(\tilde{q}\right)$. We note that in (29), the first term is viewed as the desired signal used for decoding, and the remaining two terms are the interference and the AWGN, respectively. It is easy to show that $E\{|Z_{b,r,i}{|}^{2}\}=Q||r_{2}{\left|\right|}^{2}\sigma_{b}^{2}$. For $S_{b,r,i}$ and $I_{b,r,i}$, under the condition that Q≫1, we have

(30) $$\begin{array}{cc} \; & \frac{1}{Q^{2}}{\left|S_{b,r,i}\right|}^{2}\overset{Q\to \infty }{\to }{\left|E\{|{\hat{s}}_{i}\left(\tilde{q}\right)|\}\right|}^{2}\\ = & \frac{\pi }{4}r_{2}^{H}G_{r,2}^{H}\Phi_{2}G_{r,2}\left(WW^{H}+\Sigma \right)G_{r,2}^{H}\Phi_{2}^{H}G_{r,2}r_{2}, \end{array}$$

(31) $$\begin{array}{cc} \; & \frac{1}{Q^{2}}|I_{b,r,i}{|}^{2}\leq \frac{1}{Q^{2}}{\left|{\tilde{I}}_{b,r,i}\right|}^{2}\overset{Q\to \infty }{\to }{\left|E\{|v_{i}\left(\tilde{q}\right)|\}\right|}^{2}\\ = & \frac{\pi }{4}r_{2}^{H}F_{t}\left(WW^{H}+\Sigma \right)F_{t}^{H}r_{2}, \end{array}$$

where ${\tilde{I}}_{b,r,i}≜\sum_{\tilde{q}=1}^{Q}\left|v_{i}\left(\tilde{q}\right)\right|$, and the limits are due to the law of large numbers. As a result, when Q≫1, an achievable average SINR for the DFRC-BS to decode the information-carrying symbols from the RIS is given by

(32) $$\gamma_{b,r}^{csr}=\frac{r_{2}^{H}G_{r,2}^{H}\Phi_{2}G_{r,2}\left(WW^{H}+\Sigma \right)G_{r,2}^{H}\Phi_{2}^{H}G_{r,2}r_{2}}{r_{2}^{H}F_{t}\left(WW^{H}+\Sigma \right)F_{t}^{H}r_{2}+\left(4/\pi \right)\left|\right|r_{2}{\left|\right|}^{2}\sigma_{b}^{2}/Q}.$$

If the DFRC-BS can successfully obtain $c_{i}$, namely, it satisfies that $\gamma_{b,r}^{csr}\geq \mu_{th}$, then, after performing SIC, the average sensing SINR becomes (20).

### 2.4. Problem Formulation

In previous subsections, we have analyzed the performance of the considered RIS-SR-ISAC system under the PSR and CSR settings. By jointly designing the transmit beamforming vectors, i.e., $\left\{w_{1},w_{2},\cdots ,w_{k}\right\}$, the covariance matrix of the sensing signal, i.e., Σ, the receiving beamforming vectors, i.e., $r_{1}$ and $r_{2}$, and the reflection coefficients of the RIS, i.e., Φ, it is possible to enhance the secure communications performance of the primary links, while ensuring satisfactory performances for both the sensing and the backscatter links. We formally state the problems studied in this work below.

Under the condition that the considered system works in the PSR mode, and that the DFRC-BS adopts the SWIC strategy, we consider the following sum secrecy rate maximization (SSRM) problem,

(33a) $$\begin{array}{cc} \left(P1\right)\;\;\max_{\begin{array}{c} w_{1},w_{2},\cdots ,w_{K}\\ \Sigma ,\Phi ,r_{1} \end{array}}\; & \sum_{k=1}^{K}R_{s,k}^{psr} \end{array}$$

(33b) $$\begin{array}{cc} s.t.\;\; & \gamma_{b,k}^{psr}\geq \mu_{\mathrm{th}},\forall k \end{array}$$

(33c) $$\begin{array}{cc} & \gamma_{b,t}^{\mathrm{SWIC}}\geq \gamma_{\min}, \end{array}$$

(33d) $$\begin{array}{cc} & \mathrm{Tr}\left\{W^{H}W\right\}+\mathrm{Tr}\left(\Sigma \right)\leq P_{\max}, \end{array}$$

(33e) $$\begin{array}{cc} & |\phi_{i,j}|=1,\;\forall i\in \left\{1,2\right\},j\in \left[N_{i}\right] \end{array}$$

where (33b) ensures the reliability of the RIS-based backscatter links, (33c) guarantees a sufficiently high sensing SINR, not lower than a pre-specified threshold, denoted by $\gamma_{\min}$, (33d) has the total power consumption not exceed $P_{\max}$, and (33e) represents the constant module constraint on each reflection coefficient. If the DFRC-BS adopts the ICBS strategy instead, we have the following optimization problem,

(34a) $$\begin{array}{cc} \left(P2\right)\;\;\max_{\begin{array}{c} w_{1},w_{2},\cdots ,w_{K}\\ \Sigma ,\Phi ,r_{1},r_{2} \end{array}}\; & \sum_{k=1}^{K}R_{s,k}^{psr} \end{array}$$

(34b) $$\begin{array}{cc} s.t.\;\;\;\;\;\;\;\;\; & \gamma_{b,k}^{psr}\geq \mu_{\mathrm{th}},\forall k \end{array}$$

(34c) $$\begin{array}{cc} \; & \gamma_{b,t}^{\mathrm{ICBS}}\geq \gamma_{\min}, \end{array}$$

(34d) $$\begin{array}{cc} \; & \gamma_{b,r}^{psr}\geq \mu_{\mathrm{th}}, \end{array}$$

(33d), (33e).
(34e)

It is worth noting that the key difference between problem (P1) and (P2) is that in (P2), an extra constraint on $\gamma_{b,r}^{psr}$ is imposed to ensure that the DFRC-BS can cancel all the interference from the RIS—see (34d).

Note that similar problems can be formulated for the case of CSR, which are omitted here for the sake of space limitation. We would like to point out that though the algorithms presented in next section are developed for solving (P1) and (P2) under the PSR setting, they can be easily extended to the case of CSR. We will evaluate the performance of the considered RIS-SR-ISAC system under both the PSR and the CSR settings in Section 4.

It is can be verified that both problem (P1) and (P2) are non-convex, with optimization variables highly coupled. Therefore, it is difficult to obtain their globally optimal solutions. In next section, we develop iterative algorithms to find satisfactory solutions to (P1) and (P2).

## 3. Algorithm Design for SSRM

In this section, we present our method to deal with (P1) and (P2). Since problem (P1) and (P2) have a similar mathematical structure, we mainly focus on solving (P2) in this section. We note that the presented algorithm directly adapts to (P1) by neglecting the constraint in (34d). To handle (P2), we adopt a BCA-based method. Specifically, the optimization variables in (P2) are divided into three groups, i.e., $\left\{w_{1},w_{2},\cdots ,w_{K},\Sigma \right\}$, {Φ}, and $\left\{r_{1},r_{2}\right\}$, which are optimized in an iterative manner. At each time, only one group of the optimization variables are updated while the others are fixed. For the sub-problems of updating $\left\{w_{1},w_{2},\cdots ,w_{K},\Sigma \right\}$ and {Φ}, algorithms are designed based on the semi-definite relaxation (SDR) and the successive convex approximation (SCA) methods. For the sub-problem of updating $\left\{r_{1},r_{2}\right\}$, closed-form solutions are derived. We present the details below.

### 3.1. The Sub-Problem of Optimizing $\left\{w_{1},w_{2},\cdots ,w_{K},\Sigma \right\}$

We design an SDR-based method to solve the sub-problem of optimizing $\{w_{1},w_{2},\cdots ,$$w_{K},\Sigma \}$. We first reformulate (P2) into a more tractable form. Denote $v_{1}≜{\left[\phi_{1,1},\phi_{1,2},\cdots ,\phi_{1,N_{1}}\right]}^{H}$$\in C^{N_{1}\times 1}$, $v_{2}≜{\left[\phi_{2,1},\phi_{2,2},\cdots ,\phi_{2,N_{2}}\right]}^{H}\in C^{N_{2}\times 1}$, $v≜{\left[v_{1}^{H},v_{2}^{H}\right]}^{H}$, $V_{1}≜v_{1}v_{1}^{H}$, $V_{2}≜v_{2}v_{2}^{H}$, $V≜vv^{H}$, $\bar{v}≜{\left[v^{H},1\right]}^{H}$, $\bar{V}≜\bar{v}{\bar{v}}^{H}$, $W_{k}≜w_{k}w_{k}^{H}$, $P≜\sum_{k=1}^{K}W_{k}+\Sigma$, and $P_{k}≜P-W_{k}$. Then, we can rewrite the objective function of (P2) in (34a) as

(35) $$\begin{array}{cc} \mathrm{SSR}(X,\bar{V})= & \sum_{k=1}^{K}\sum_{j=1}^{2}\frac{1}{2}[\log_{2}\left({\bar{A}}_{k}\right)+\log_{2}\left({\bar{B}}_{j}^{\left(e\right)}\right)\\ \; & \;\;\;-\log_{2}\left({\bar{B}}_{k}\right)-\log_{2}\left({\bar{A}}_{k,j}^{\left(e\right)}\right)], \end{array}$$

where $X≜\{W_{1},W_{2},\cdots ,W_{K},\Sigma \}$, and

(36) $$\begin{array}{cc} {\bar{A}}_{k} & ≜\mathrm{Tr}\left(\Xi_{1,k}P\Xi_{1,k}^{H}\bar{V}\right)+\mathrm{Tr}\left(\Xi_{2,k}P\Xi_{2,k}^{H}\bar{V}\right)+\sigma_{k}^{2}, \end{array}$$

(37) $$\begin{array}{cc} {\bar{B}}_{k} & ≜\mathrm{Tr}\left(\Xi_{1,k}P_{k}\Xi_{1,k}^{H}\bar{V}\right)+\mathrm{Tr}\left(\Xi_{2,k}P\Xi_{2,k}^{H}\bar{V}\right)+\sigma_{k}^{2}, \end{array}$$

(38) $$\begin{array}{cc} {\bar{A}}_{k,j}^{\left(e\right)} & ≜\mathrm{Tr}\left(\Xi_{j,e}\left(W_{k}+\Sigma \right)\Xi_{j,e}^{H}\bar{V}\right)+\sigma_{e}^{2}, \end{array}$$

(39) $$\begin{array}{cc} {\bar{B}}_{j}^{\left(e\right)} & ≜\mathrm{Tr}\left(\Xi_{j,e}\Sigma \Xi_{j,e}^{H}\bar{V}\right)+\sigma_{e}^{2}, \end{array}$$

with $\Xi_{1,e}≜{\left[G_{r,1}^{H}\mathrm{diag}\left(g_{e,1}\right),G_{r,2}^{H}\mathrm{diag}\left(g_{e,2}\right),h_{e}\right]}^{H}\in C^{(N+1)\times M}$, and $\Xi_{2,e}≜[G_{r,1}^{H}\mathrm{diag}\left(g_{e,1}\right),$$-G_{r,2}^{H}\mathrm{diag}\left(g_{e,2}\right),h_{e}{]}^{H}\in C^{(N+1)\times M}$, $\Xi_{1,k}≜{\left[G_{r,1}^{H}\mathrm{diag}\left(g_{k,1}\right),0_{M\times N_{2}},h_{k}\right]}^{H}\in C^{(N+1)\times M}$ and $\Xi_{2,k}≜{\left[0_{M\times N_{1}}G_{r,2}^{H}\mathrm{diag}\left(g_{k,2}\right),0_{M\times 1}\right]}^{H}\in C^{(N+1)\times M}$.

Based on (35), with $\bar{V}$ and $\left\{r_{1},r_{2}\right\}$ fixed, it can be shown that (P2) is equivalent to the following problem,

(40a) $$\begin{array}{cc} \max_{X}\; & \mathrm{SSR}(X,\bar{V}) \end{array}$$

(40b) $$\begin{array}{cc} s.t.\; & \mathrm{Tr}\left(\Xi_{4,k}W_{k}\right)\geq \mu_{th}\left(\mathrm{Tr}\left(\Xi_{5,k}P_{k}\right)+\sigma_{k}^{2}\right),\forall k, \end{array}$$

(40c) $$\begin{array}{cc} \; & \mathrm{Tr}\left(\Xi_{6}P\right)\geq \mu_{th}\left(\mathrm{Tr}\left(\Xi_{7}P\right)+\sigma_{b1}^{2}\right), \end{array}$$

(40d) $$\begin{array}{cc} \; & \mathrm{Tr}\left(\Xi_{8}P\right)\geq \gamma_{min}\sigma_{b2}^{2}, \end{array}$$

(40e) $$\begin{array}{cc} \; & \mathrm{Tr}\left(P\right)\leq P_{\max}, \end{array}$$

(40f) $$\begin{array}{cc} \; & \mathrm{Rank}\left(W_{k}\right)=1, \end{array}$$

where $\Xi_{3,k}≜\Xi_{1,k}^{H}\bar{V}\Xi_{1,k}$, $\Xi_{4,k}≜\Xi_{2,k}^{H}\bar{V}\Xi_{2,k}$, $\Xi_{5,k}≜\Xi_{3,k}+\Xi_{4,k}$, $\Xi_{6}≜U_{r}^{H}\bar{V}U_{r}$, $U_{r}≜{\left[0_{M\times N_{1}},G_{r,2}^{H}\mathrm{diag}\left(G_{r,2}r_{2}\right),0_{M\times 1}\right]}^{H}\in C^{(N+1)\times M}$, $\Xi_{7}≜F_{t}^{H}r_{2}r_{2}^{H}F_{t}$, $\Xi_{8}≜F_{t}^{H}r_{1}r_{1}^{H}F_{t}$, $\sigma_{b1}^{2}≜\sigma_{b}^{2}\mathrm{Tr}\left(r_{2}r_{2}^{H}\right)$, and $\sigma_{b2}^{2}≜\sigma_{b}^{2}\mathrm{Tr}\left(r_{1}r_{1}^{H}\right)$.

We note that the main difficulty in solving (40) lies in the non-concavity of $\mathrm{SSR}(X,\bar{V})$ with respect to X. The following lemma—see, e.g., ref. ([50], Lemma 1) and ([51], Lemma 1)—is exploited to transform it into a more tractable form.

**Lemma** **1.** *For ∀x>0, let φ(t)=−tx+lnt+1. Then, the following equation holds,*

(41) $$\begin{array}{c} -\ln x=\max_{t>0}\varphi \left(t\right), \end{array}$$

*where at the right-hand-side of* (41)*, the maximum is achieved at* t=1/x.

Based on Lemma 1, $\mathrm{SSR}(X,\bar{V})$ can be written as the following equivalent form,

(42) $$\begin{array}{cc} \mathrm{SSR}(X,\bar{V})= & \max_{T}\;\bar{\mathrm{SSR}}\left(X,\bar{V},T\right), \end{array}$$

(43) $$\begin{array}{cc} \bar{\mathrm{SSR}}\left(X,\bar{V},T\right)= & \frac{1}{2\ln2}\sum_{k=1}^{K}\sum_{j=1}^{2}[\psi_{k}\left(X,\bar{V},t_{k}\right)\\ \; & \;\;\;\;\;-\psi_{k,j}^{\left(e\right)}\left(X,\bar{V},t_{k,j}^{\left(e\right)}\right)], \end{array}$$

where $T≜\left\{t,t_{1}^{\left(e\right)},t_{2}^{\left(e\right)}\right\}$, $t≜{\left[t_{1},t_{2},\cdots ,t_{K}\right]}^{T}$, $t_{j}^{\left(e\right)}≜{\left[t_{1,j}^{\left(e\right)},t_{2,j}^{\left(e\right)},\cdots ,t_{K,j}^{\left(e\right)}\right]}^{T}$ for ∀j∈{1,2}, and

(44) $$\begin{array}{cc} \psi_{k}\left(X,\bar{V},t_{k}\right)≜ & \ln\left({\bar{A}}_{k}\right)-t_{k}{\bar{B}}_{k}+\ln t_{k}+1, \end{array}$$

(45) $$\begin{array}{cc} \psi_{k,j}^{\left(e\right)}\left(X,\bar{V},t_{k,j}^{\left(e\right)}\right)≜ & t_{k,j}^{\left(e\right)}{\bar{A}}_{k,j}^{\left(e\right)}-\ln t_{k,j}^{\left(e\right)}-\ln\left({\bar{B}}_{j}^{\left(e\right)}\right)-1. \end{array}$$

As a result, problem (40) is actually equivalent to the following problem,

(46a) $$\begin{array}{cc} \max_{X,T}\; & \bar{\mathrm{SSR}}\left(X,\bar{V},T\right) \end{array}$$

s.t.   (40b), (40c), (40d), (40e), (40f).
(46b)

We solve (46) by alternating between updating X with T fixed and updating T with X. According to Lemma 1, with X fixed, the optimal value of T is given by

(47) $$\begin{array}{c} t_{k}=\frac{1}{{\bar{B}}_{k}},\;t_{k,j}^{\left(e\right)}=\frac{1}{{\bar{A}}_{k,j}^{\left(e\right)}},\;\;\forall k,j. \end{array}$$

With T fixed, (46) is still non-convex due to the rank-one constraints in (40f). To deal with it, we follow the idea of SDR [52,53]. Specifically, we neglect the rank-one constraints, and consequently, (46) becomes a convex semi-definite programming (SDP) problem, which can be efficiently solved using a convex optimization toolbox, such as CVX [54]. We iteratively and alternatingly update T and X until they converge. It is worth noting that the ultimately obtained $W_{k}$ may not be rank-one. If this is the case, we recover a rank-one solution by using the Gaussian randomization method [30,52]. In addition, an alternative low-complexity method to obtain a rank-one is to perform eigenvalue decomposition. Specifically, for ∀k∈[K], we set $w_{k}=\sqrt{\lambda_{\max}\left(W_{k}\right)}e_{\max}\left(W_{k}\right)$.

### 3.2. The Sub-Problem of Optimizing Φ

In this subsection, we handle the sub-problem of optimizing Φ. Recall that Φ is a diagonal matrix satisfying that Φ=diag{v}, and that $\bar{v}={\left[v^{H},1\right]}^{H}$. With X and $\left\{r_{1},r_{2}\right\}$ fixed, the sub-problem of optimizing Φ can be equivalently written as

(48a) $$\begin{array}{cc} \max_{\bar{V}⪰0}\; & \mathrm{SSR}(X,\bar{V})\\ s.t.\;\; & (40b),(40c), \end{array}$$

(48b) $$\begin{array}{cc} \; & \bar{V}\left[n,n\right]=1,\forall n\in \left[N+1\right], \end{array}$$

(48c) $$\begin{array}{cc} \; & \mathrm{Rank}(\bar{V})=1, \end{array}$$

where instead of directly optimizing Φ, we optimize $\bar{V}$ first. After (48) is solved, we recover a satisfactory Φ based on the obtained $\bar{V}$.

Due to the negative logarithm terms in (35), $\mathrm{SSR}(X,\bar{V})$ is not concave with respect to $\bar{V}$. To handle this issue, based on the idea of SCA [55], we linearize those negative logarithm terms. Specifically, for ∀(k,j), we replace $-\log_{2}\left({\bar{B}}_{k}\right)$ and $-\log_{2}\left({\bar{A}}_{k,j}^{\left(e\right)}\right)$ with their first order Taylor expansions at some point, denoted by $\tilde{V}$, and obtain the following function, which serves as a concave surrogate function of $\mathrm{SSR}(X,\bar{V})$,

(49) $$\begin{array}{cc} \tilde{\mathrm{SSR}}\left(\bar{V},\tilde{V}\right)= & \sum_{k=1}^{K}\sum_{j=1}^{2}\frac{1}{2\ln2}[\ln\left({\bar{A}}_{k}\right)+\ln\left({\bar{B}}_{j}^{\left(e\right)}\right)\\ \; & \;\;\;-\ln\left({\tilde{B}}_{k}\right)-\ln\left({\tilde{A}}_{k,j}^{\left(e\right)}\right)\\ \; & \;\;\;-\mathrm{Tr}\left(D_{k,j}\left(\bar{V}-\tilde{V}\right)\right)], \end{array}$$

where ${\tilde{B}}_{k}≜\mathrm{Tr}\left(\Xi_{1,k}P_{k}\Xi_{1,k}^{H}\tilde{V}\right)+\mathrm{Tr}\left(\Xi_{2,k}P\Xi_{2,k}^{H}\tilde{V}\right)+\sigma_{k}^{2}$, ${\tilde{A}}_{k,j}^{\left(e\right)}≜\mathrm{Tr}\left(\Xi_{j,e}\left(W_{k}+\Sigma \right)\Xi_{j,e}^{H}\tilde{V}\right)+\sigma_{e}^{2}$, and $D_{k,j}≜\frac{1}{{\tilde{B}}_{k}}\left(\Xi_{1,k}P_{k}\Xi_{1,k}^{H}+\Xi_{2,k}P\Xi_{2,k}^{H}\right)+\frac{1}{{\tilde{A}}_{k,j}^{\left(e\right)}}\Xi_{j,e}\left(W_{k}+\Sigma \right)\Xi_{j,e}^{H}$. By replacing $\mathrm{SSR}(X,\bar{V})$ with $\tilde{\mathrm{SSR}}\left(\bar{V},\tilde{V}\right)$ in (48a), and neglecting the rank-one constraint in (48c), we obtain the following problem,

(50a) $$\begin{array}{cc} \max_{\bar{V}⪰0}\; & \tilde{\mathrm{SSR}}\left(\bar{V},\tilde{V}\right) \end{array}$$

s.t.   (40b), (40c), (48b),
(50b)

which is a convex SDP problem, and can be efficiently solved by using CVX [54]. Following the SCA method in [55], we repeatedly solve (50), and at each time, $\tilde{V}$ is updated as the obtained optimal $\bar{V}$. Then, the optimal solution to (50) will converge to a Karush–Kuhn–Tucker solution to problem (48) in the absence of the rank-one constraint. It is also worth noting that in general, the obtained $\bar{V}$ is not a rank-one matrix, and therefore, we adopt the Gaussian randomization method in [30,52] to recover a satisfactory rank-one solution.

### 3.3. The Sub-Problem of Optimizing $\left\{r_{1},r_{2}\right\}$

In this subsection, we optimize $\left\{r_{1},r_{2}\right\}$ with $\left\{w_{1},w_{2},\cdots ,w_{K},\Sigma \right\}$ and Φ fixed. Since $r_{1}$ only appears in constraint (34c), and $r_{2}$ only appears in constraint (34d), the optimization of $\left\{r_{1},r_{2}\right\}$ can be formulated as two separate problems as follows,

(51) $$\begin{array}{cc} \max_{r_{1}} & \frac{r_{1}^{H}\Omega_{r}r_{1}}{\sigma_{b}^{2}r_{1}^{H}r_{1}}, \end{array}$$

(52) $$\begin{array}{cc} \max_{r_{2}} & \frac{r_{2}^{H}\Omega_{r,1}r_{2}}{r_{2}^{H}\Omega_{r,2}r_{2}}, \end{array}$$

where $\Omega_{r}≜F_{t}PF_{t}^{H}$, and

(53a) $$\begin{array}{cc} \Omega_{r,1} & ≜G_{r,2}^{H}\Phi_{2}G_{r,2}PG_{r,2}^{H}\Phi_{2}^{H}G_{r,2},\\ \; & =G_{r,2}^{H}\left(V_{2}⊙\left(G_{r,2}PG_{r,2}^{H}\right)\right)G_{r,2}, \end{array}$$

(53b) $$\begin{array}{cc} \Omega_{r,2} & ≜\sigma_{b}^{2}I_{M}+F_{t}PF_{t}^{H}. \end{array}$$

Since (51) is a Rayleigh quotient problem [16], its optimal solution is directly written as

(54) $$\begin{array}{c} r_{1,opt}=e_{\max}\left(\Omega_{r}\right). \end{array}$$

For problem (52), according to [56], it is a generalized Rayleigh quotient problem, and its optimal solution is given by

(55) $$\begin{array}{c} r_{2,opt}=e_{\max}\left(\Omega_{r,2}^{-1}\Omega_{r,1}\right). \end{array}$$

We summarize the detailed steps of the proposed BCA-based algorithm in Algorithm 1, wherein we iteratively update X, $\bar{V}$, and $\left\{r_{1},r_{2}\right\}$ for a total number of $\tau_{\max}$ times. For the sub-problem of updating X and the sub-problem of updating $\bar{V}$, $\tau_{1,\max}$ and $\tau_{2,\max}$ iterations are conducted, respectively.
**Algorithm 1** BCA-based algorithm  1:Initialize X, Φ, $r_{1}$ and $r_{2}$.  2:**Repeat** for $\tau_{\max}$ times  3:    **Repeat** for $\tau_{1,\max}$ times  4:        Update X by solving (46) with T;  5:        Update T according to (47);  6:    **End**  7:    **Repeat** for $\tau_{2,\max}$ times  8:        Set $\tilde{V}=\bar{V}$;  9:        Update $\bar{V}$ by solving (50);10:  **End**11:  Update $r_{1}$ according to (54);12:  Update $r_{2}$ according to (55);13:**End**14:Recover a rank-one solution using the method of Gaussian randomization.

We analyze the computational complexity of Algorithm 1 above. In fact, the computation burden of Algorithm 1 mainly stems from the steps for updating X in line 3–6, and the steps for updating $\bar{V}$ in line 7–10. To update X, the convex SDP in (46) is solved for a total number of $\tau_{1,\max}$ times, and the corresponding computational complexity is $O\left(\tau_{1,max}\cdot \left(\max{\left\{M,K\right\}}^{4}M^{\frac{1}{2}}\right)\right)\log\left(\epsilon^{-1}\right)$, where ϵ is the accuracy tolerance [57]. To update $\bar{V}$, the convex SDP in (50) is solved for a total number of $\tau_{2,\max}$ times, and the corresponding computational complexity is $O\left(\tau_{2,max}\cdot \left({\left(N+K\right)}^{4}N^{\frac{1}{2}}\right)\right)\log\left(\epsilon^{-1}\right)$. Therefore, the overall computational complexity of Algorithm 1 is about $O\left(\tau_{max}\tau_{1,max}\left(\max{\left\{M,K\right\}}^{4}M^{\frac{1}{2}}\right)+\tau_{max}\tau_{2,max}\left({\left(N+K\right)}^{4}N^{\frac{1}{2}}\right)\right)\log\left(\epsilon^{-1}\right)$.

In summary, in this section, we have presented a BCA-based method to handle problem (P2). We note that Algorithm 1 adapts to (P1) by neglecting the constraint in (34d). In the next section, we evaluate the performance of the considered RIS-SR-ISAC system.

## 4. Simulation Results

In this section, simulation results are presented to evaluate the performance of the considered RIS-SR-ISAC system.

In our simulations, the DFRC-BS, the RIS, the sensing target, two different UTs, and Eve are distributed in a two-dimensional plane whose locations are $l_{b}=\left(0,0\right)$, $l_{r}=\left(10,20\right)$, $l_{t}=\left(-10,-50\right)$, $l_{u_{1}}=\left(0,70\right)$, $l_{u_{2}}=\left(0,60\right)$, and $l_{e}=\left(0,50\right)$, respectively. We model the distance-based path-loss as $L_{0}d_{i,j}^{-\beta_{i,j}}$ for $\forall i,j\in \{b,r,t,u_{1},u_{2},e\}$ where $L_{0}$ is the path-loss at a distance of 1 m, $d_{i,j}=\left|\right|l_{i}-l_{j}\left|\right|$ is the link distance, and $\beta_{i,j}$ is the path-loss exponent. We set $L_{0}=-20$ (dB), $\beta_{r,u_{1}}=\beta_{r,u_{2}}=\beta_{b,r}=\beta_{b,t}=\beta_{r,e}=2$, and $\beta_{b,u_{1}}=\beta_{b,u_{2}}=\beta_{b,e}=4$. And unless specified, we set the number of the antennas at the DFRC-BS as M=8, the number of the RUs at the RIS as N=20 with $N_{1}=10$, the power budget at the DFRC-BS as $P_{\max}=40$ dBm, and the noise power as $\sigma_{1}^{2}=\sigma_{2}^{2}=\sigma_{b}^{2}=\sigma_{e}^{2}=-70$ dBm. The channel between the target and the DFRC-BS is modeled as a light-of-sight channel. Other channels are considered to be Rician distributed, and we set the Rician factor as 3. The requirement of the sensing SINR is set as $\gamma_{\min}=10$ dB, and the SINR threshold for decoding the data from the RIS is set as $\mu_{th}=5$ dB for the case of PSR and $\mu_{th}=8$ dB for the case of CSR. Furthermore, under the CSR case, we set Q=64. Moreover, the following three baseline schemes are presented for comparison:

1. Baseline 1: All the RUs of the RIS are used to achieve backscatter communications, namely, $N_{1}=0$.
2. Baseline 2: The RIS purely assists the secure transmissions from the FDRC-BS to the UTs, namely, $N_{2}=0$.
3. Baseline 3: The RIS adopts randomly generated phase shifts without optimization.

**Figure 2:**
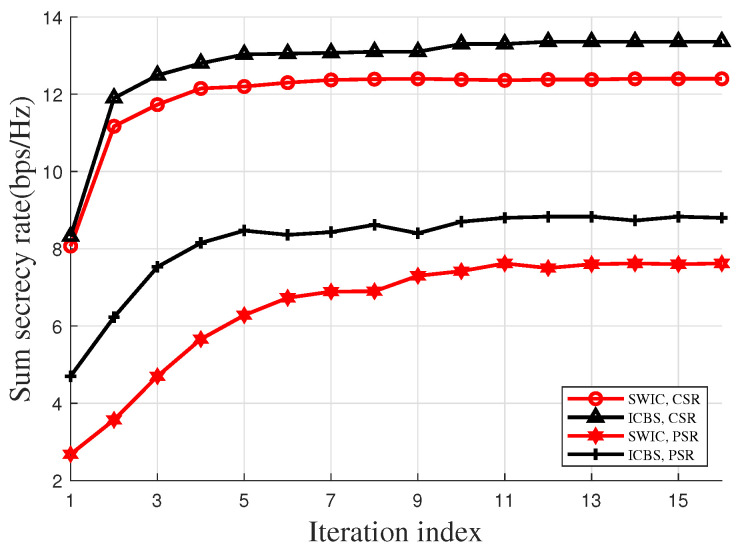
Convergence performance of the proposed algorithm.

In Figure 2, we evaluate the convergence of the proposed algorithm. It can be observed from Figure 2 that under each case, as the iteration goes, the sum secrecy rate exhibits a non-decreasing trend and ultimately converges to a limit value. Moreover, Figure 2 also reveals that the proposed algorithm can return a satisfactory solution within a small number of iterations. In particular, under both the PSR and the CSR settings, 13 iterations are enough for convergence.

**Figure 3:**
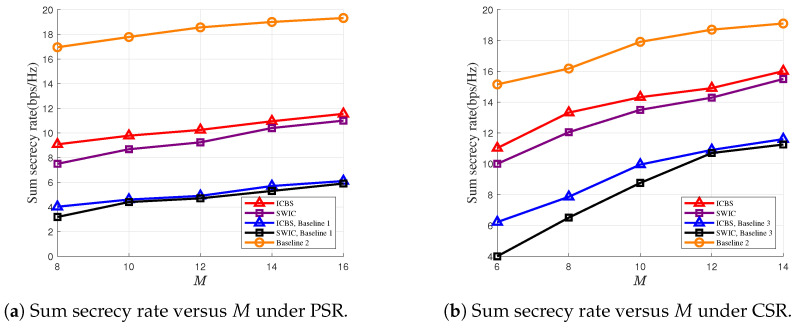
Sum secrecy rate versus the number of DFRC-BS antennas under PSR and CSR cases.

In Figure 3a,b, we plot the sum secrecy rates versus the numbers of the antennas at the DFRC-BS under PSR and CSR settings, respectively. It can be observed that the sum secrecy rates increase with the number of the antennas at the DFRC-BS, i.e., *M*, thanks to the increasing spatial degree-of-freedom at the DFRC-BS. For the PSR case, Figure 3a shows that the sum secrecy rates of the proposed scheme are higher than the scheme wherein all the RUs of the RIS are used for backscatter communications. This is because in the latter scheme, the RIS becomes incapable of enhancing the strength of the signal from the DFRC-BS, and the signal reflected by the RIS only results in interference that degrades the signal quality of the primary links. We also note that compared with the situation where the RIS is purely used to assist the primary transmissions, the sum secrecy rates are decreased if the RIS is used to establish a backscatter link. Figure 3a,b reveal that such a decrease is more severe in the case of PSR, and CSR achieves a better secure communications performance.

**Figure 4:**
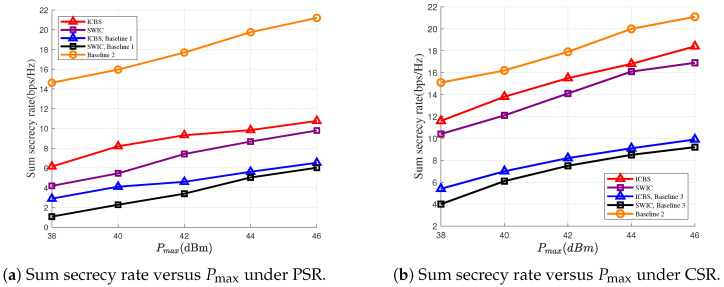
Sum secrecy rate versus the maximum transmit power of the DFRC-BS under PSR and CSR cases.

In Figure 4a,b, we illustrate the sum secrecy rates versus the maximum transmit powers of the DFRC-BS under the PSR and the CSR settings, respectively. Figure 4a,b reveal that the increase of the transmit power leads to an improved secure communication performance between the DFRC-BS and the UTs. Figure 4a,b also demonstrate that compared with directly treating the backscatter signal from the RIS as noise, performing SIC is beneficial. For example, at the point with $P_{\max}=44$ dBm, the secrecy rate can be increased by about 13% under the PSR setting and 5% under the CSR setting.

**Figure 5:**
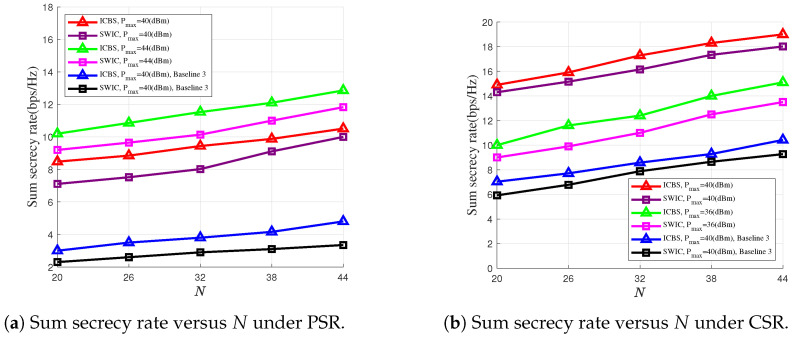
Sum secrecy rate versus the number of RUs under PSR and CSR cases.

In Figure 5a,b, we plot the sum secrecy rates versus the numbers of the RUs at the RIS under the PSR and the CSR cases, respectively, where we set $N_{2}=10$ and M=10. From Figure 5a,b, we can see that as *N* increases, the secrecy rates also increase, thanks to the enhanced strength of the signal reflected by the RIS. This demonstrates the advantage of using an increasing number of the RUs. For both the PSR and the CSR cases, Figure 5a,b also show that compared with the case where the RIS adopts randomly generated phase shifts, the secure communications performance can be significantly improved after a dedicated optimization procedure, which highlights the effectiveness of the proposed optimization algorithm.

**Figure 6:**
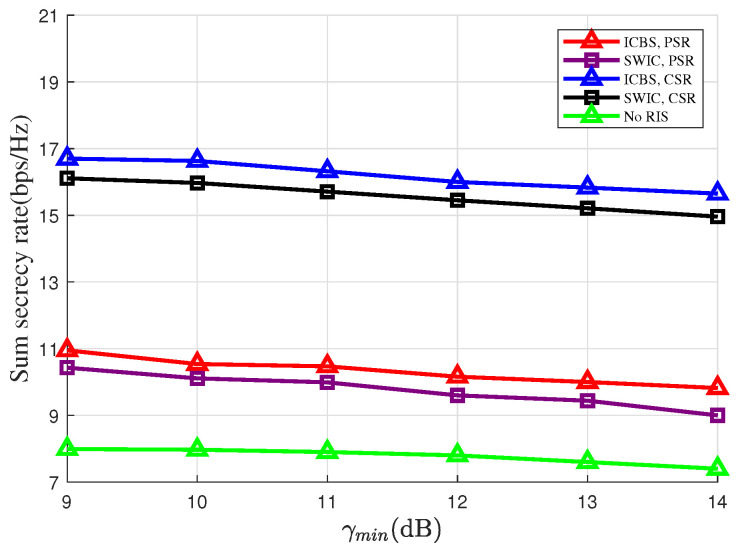
Sum secrecy rate versus the SINR threshold $\gamma_{\min}$ of target sensing.

In Figure 6, we plot the sum secrecy rates versus the thresholds of the sensing SINR, i.e., $\gamma_{\min}$, where we set M=10 and $N_{1}=20$. From Figure 6, a trade-off relationship between the sensing and the secure communications performance can be observed. Specifically, the increase of $\gamma_{\min}$ inevitably leads to the decrease of the sum secrecy rate. In fact, to improve the sensing performance, careful control of the interference due to the presence of the backscatter link is required. Figure 6 also shows that under both the PSR and the CSR cases, the sum secrecy rates under the ICBS strategy are higher than that under the SWIC strategy. In fact, removing the interference from the RIS makes the requirement of the sensing SINR a looser constraint. This enables the DFRC-BS to allocate more power to improve the secrecy rates of the primary links. As a result, by adopting the ICBS strategy, the secure performance of the primary link can be significantly improved. Moreover, compared with the case where there is no RIS in the considered system, the sum secrecy rate can be significantly improved if the reflection coefficients are delicately optimized. This indicates that though in the RIS-SR-ISAC system the RIS-based backscatter link will interfere with the sensing link, the RIS can still provide substantial performance gain.

**Figure 7:**
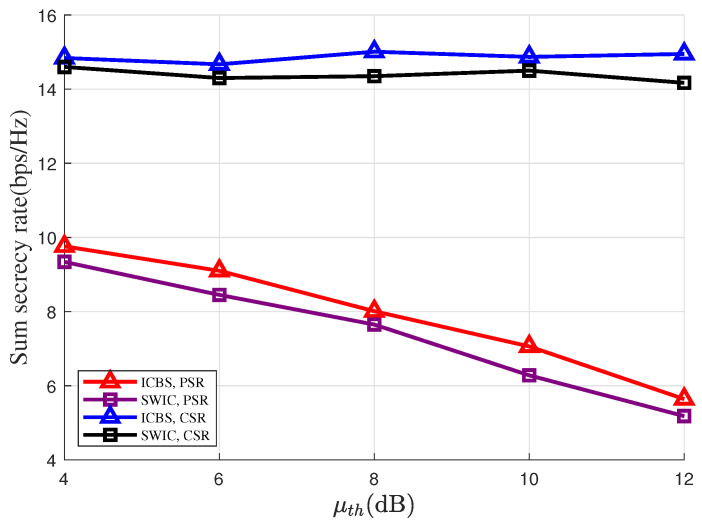
Sum secrecy rate versus SINR threshold $\mu_{th}$ of the RIS-based backscatter link.

In Figure 7, we plot the sum secrecy rates versus the SINR thresholds of the RIS-based backscatter link, i.e., $\mu_{th}$, where we set M=10. It is interesting to note that compared with the case of PSR, the sum secrecy rates under the CSR setting are less sensitive to the value of $\mu_{th}$. Specifically, for the PSR case, as $\mu_{th}$ increases from 4 to 12 dB, the sum secrecy rates get decreased by about 4 bps/Hz. Whereas for the CSR case, there is no significant change in the sum secrecy rates as $\mu_{th}$ increases from 4 to 12 dB. This is because under the CSR setting, for each information-carrying symbol from the RIS, multiple signal samples can be combined together to perform decoding, which significantly boosts the SINR of the backscatter link.

## 5. Conclusions

This work has been focused on designing effective secure transmission schemes for RIS-SR-ISAC systems. Under both the PSR and the CSR settings, we aim to maximize the sum secrecy rate of the primary links under a group of QoS constraints on the backscatter and the sensing links. Two different strategies have been considered for the DFRC-BS to handle the interference from the backscatter link, namely the SWIC and the ICBS strategies. BCA-based algorithms have been designed to handle the established non-convex problems. Our simulation results have verified the effectiveness of the proposed algorithm, and have revealed that performing interference cancellation at the DFRC-BS can effectively improve the secure communications performance of the considered RIS-SR-ISAC system.

## Figures and Tables

**Table 1:** Comparison.

Publication	Security	RIS	ISAC	SR
[22]	✓	×	×	✓
[23]	✓	✓	×	✓
[31]	×	✓	×	✓
[32]	×	✓	✓	×
[28]	✓	✓	✓	×
[30]	✓	✓	✓	×
[33]	×	×	✓	×
[15]	×	✓	✓	✓
[16]	×	✓	✓	✓
[17]	×	✓	✓	✓
Our work	✓	✓	✓	✓

## Data Availability

The original contributions presented in this study are included in the article. Further inquiries can be directed to the corresponding author.
